# Clonal memory in human embryonic stem cells biases fate potential during endoderm differentiation

**DOI:** 10.64898/2026.09.02.748863

**Published:** 2026-09-03

**Authors:** Madeleine Linneberg-Agerholm, Álvaro Bustos Gutiérrez, Stine Lind Hansen, Yutaka Shimizu, Nicolaj Strøyer Christophersen, Samantha A. Morris, Atefeh Lafzi

## Abstract

Cell fate decisions during development are shaped not only by extrinsic signals but also by heritable intrinsic states passed on across cell division. The extent to which this phenomenon, termed clonal memory, can explain the persistent heterogeneity observed from directed differentiation of human embryonic stem cells is unclear. Here, we combine lineage tracing with single-cell transcriptomics and chromatin accessibility profiling to track clonal behaviour across human embryonic stem cell differentiation towards definitive endoderm. Using a lentiviral barcoding system coupled with a split-well sampling strategy, we find that clonally related cells exhibit reproducible, probabilistic fate outcomes that cannot be explained by signalling environment alone. Fate-biased clones are transcriptionally indistinguishable at the pluripotent stage yet display distinct chromatin accessibility landscapes at lineage-specific cis-regulatory elements. Pre-existing accessibility at these lineage-specific regulatory regions distinguish clones that undergo successful endoderm differentiation from those that generate off-target mesoderm derivatives. Together, these findings provide an explanation for how off-target populations arise during directed differentiation, identifying heritable chromatin states within pluripotent cultures as a source of variability relevant to stem cell-derived *in vitro* models and cell therapies.

## Full Text Availability

The license terms selected by the author(s) for this preprint version do not permit archiving in PMC. The full text is available from the preprint server.

